# Introduction to the LIVECAT web-based computerized adaptive testing platform

**DOI:** 10.3352/jeehp.2020.17.27

**Published:** 2020-09-29

**Authors:** Dong Gi Seo, Jeongwook Choi

**Affiliations:** 1 Department of Psychology, College of Social Sciences & Hallym Applied Psychology Institute, Hallym University, Chuncheon, Korea; 2 The CAT Korea Company, Seoul, Korea; Hallym University, Korea

**Keywords:** Computerized adaptive testing, Item response theory, Likelihood functions, Psychometrics, Software

## Abstract

This study introduces LIVECAT, a web-based computerized adaptive testing platform. This platform provides many functions, including writing item content, managing an item bank, creating and administering a test, reporting test results, and providing information about a test and examinees. The LIVECAT provides examination administrators with an easy and flexible environment for composing and managing examinations. It is available at http://www.thecatkorea.com/. Several tools were used to program LIVECAT, as follows: operating system, Amazon Linux; web server, nginx 1.18; WAS, Apache Tomcat 8.5; database, Amazon RDMS—Maria DB; and languages, JAVA8, HTML5/CSS, Javascript, and jQuery. The LIVECAT platform can be used to implement several item response theory (IRT) models such as the Rasch and 1-, 2-, 3-parameter logistic models. The administrator can choose a specific model of test construction in LIVECAT. Multimedia data such as images, audio files, and movies can be uploaded to items in LIVECAT. Two scoring methods (maximum likelihood estimation and expected a posteriori) are available in LIVECAT and the maximum Fisher information item selection method is applied to every IRT model in LIVECAT. The LIVECAT platform showed equal or better performance compared with a conventional test platform. The LIVECAT platform enables users without psychometric expertise to easily implement and perform computerized adaptive testing at their institutions. The most recent LIVECAT version only provides a dichotomous item response model and the basic components of CAT. Shortly, LIVECAT will include advanced functions, such as polytomous item response models, weighted likelihood estimation method, and content balancing method.

## Introduction

### Background/rationale

Computerized adaptive testing (CAT) has been used widely for licensing/certification examinations. Many institutions in the United States administer CAT-based license tests for health professionals, including the National Council of State Boards of Nursing for the National Council Licensure Examination for Registered Nurses; the American Society for Clinical Pathology Board of Certification for 24 examinations, including the medical laboratory scientist, medical laboratory technician, phlebotomy technician, and histotechnician examinations; the National Registry of Emergency Medical Technicians (EMTs) for the emergency medical responder, EMT, and advanced-EMT exams; and the National Association of Boards of Pharmacy for the North American Pharmacist Licensure Examination. In Korea, a few institutions have become interested in administering their examinations using CAT. In particular, the Korea Health Personnel Licensing Examination Institute has prepared to implement CAT for the Korean Medical Licensing Examination [1]. Although interest in CAT has gradually increased, few CAT platforms for licensing/certification examinations have been developed in South Korea. For this reason, a web-based CAT platform was developed for licensing/certification examinations, including educational/psychological tests.

### Objectives

This study introduces LIVECAT, a web-based CAT platform available at http://www.thecatkorea.com/. Its development tools, execution algorithm, functions, and user interface are presented below.

## Two LIVECAT interfaces

LIVECAT provides examination administrators with an easy and flexible environment for composing and managing examinations. It also stores responses and can track changes in respondents. LIVECAT provides 2 testing forms: computer-based testing and CAT. Examinees can access tests administered using LIVECAT through a variety of internet-connected devices (desktops, laptops, smartphones, and tablet computers).

### Development tools

Several tools were used for programming LIVECAT, as follows: (1) operating system, Amazon Linux; (2) web server, nginx 1.18; (3) WAS, Apache Tomcat 8.5; (4) database, Amazon RDMS—Maria DB; and (5) languages, JAVA8, HTML5/CSS, Javascript, and jQuery.

### Item response theory models

Several item response theory (IRT) models were implemented in the LIVECAT platform, as follows: (1) Rasch model, (2) 1-parameter logistic model, (3) 2-parameter logistic model, and (4) 3-parameter logistic model. The administrator can choose a specific model for test construction in LIVECAT.

## Diagram of the LIVECAT algorithm

**Fig. 1.**
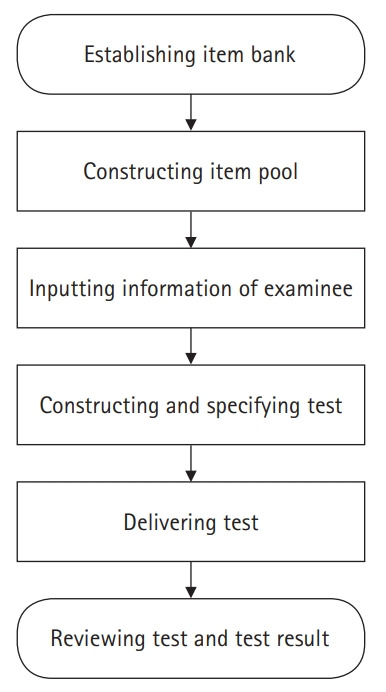
Flowchart of the process of constructing a test in LIVECAT.

**Fig. 2.**
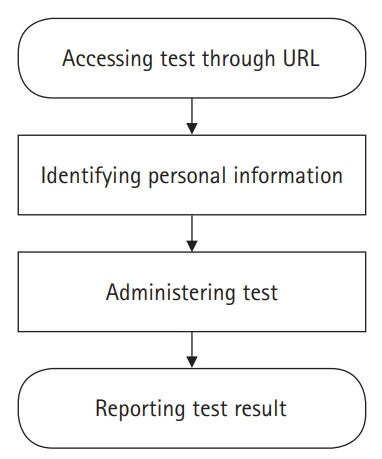
Flowchart of the process of taking a test in LIVECAT.

**Fig. 3.**
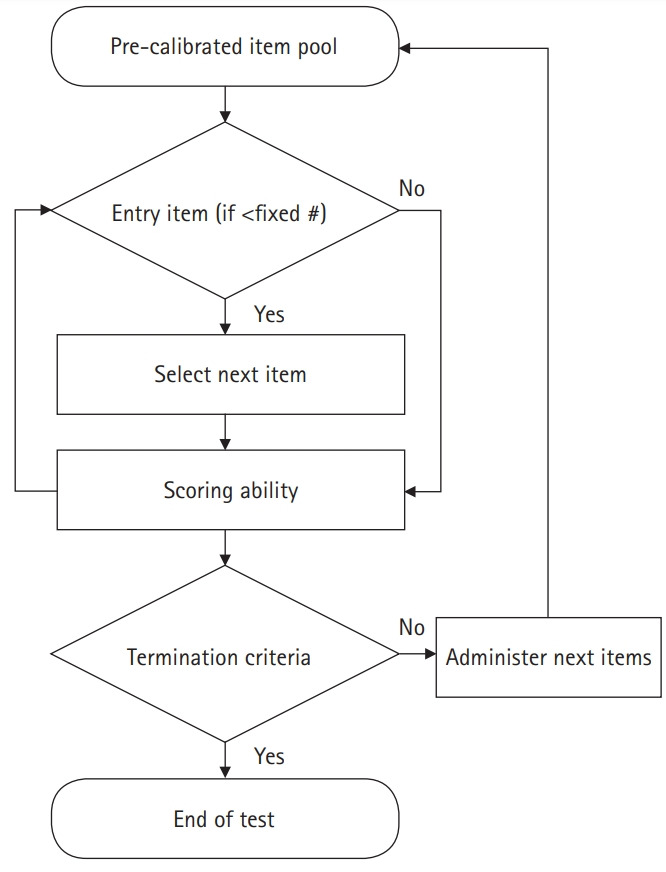
The computerized adaptive testing algorithm used to estimate an examinee’s ability.

Fig. 1 presents the process of constructing a test in the administrator account on LIVECAT. Fig. 2 shows the process of taking a test using an examinee account on LIVECAT. Fig. 3 is a flowchart of the CAT algorithm used to estimate an examinee’s ability in LIVECAT [2]. The CAT algorithm is embedded in the “administering test” section, as shown in Fig. 2, and several IRT models and rules of the CAT algorithm can be selected in the “constructing and specifying test” construction, as shown in Fig. 1.

### Establishment of the item bank

**Fig. 4.**
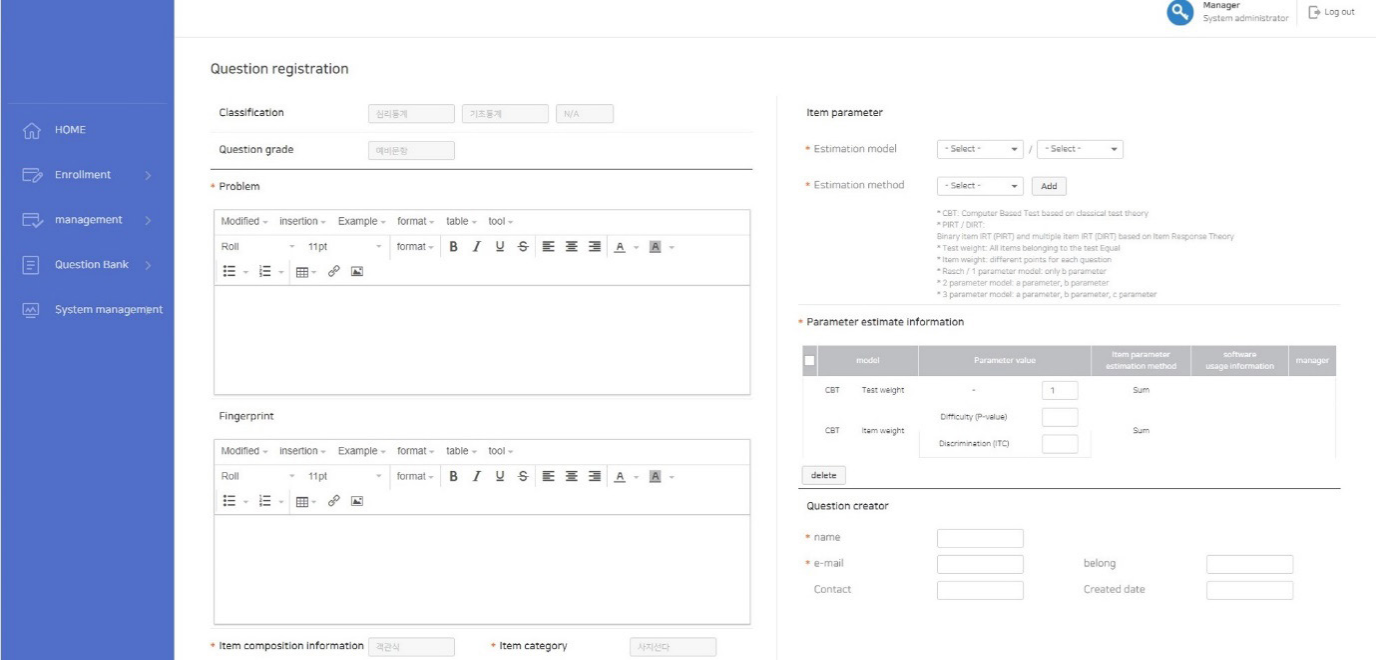
Screenshot of the input for items and item parameters.

**Fig. 5.**
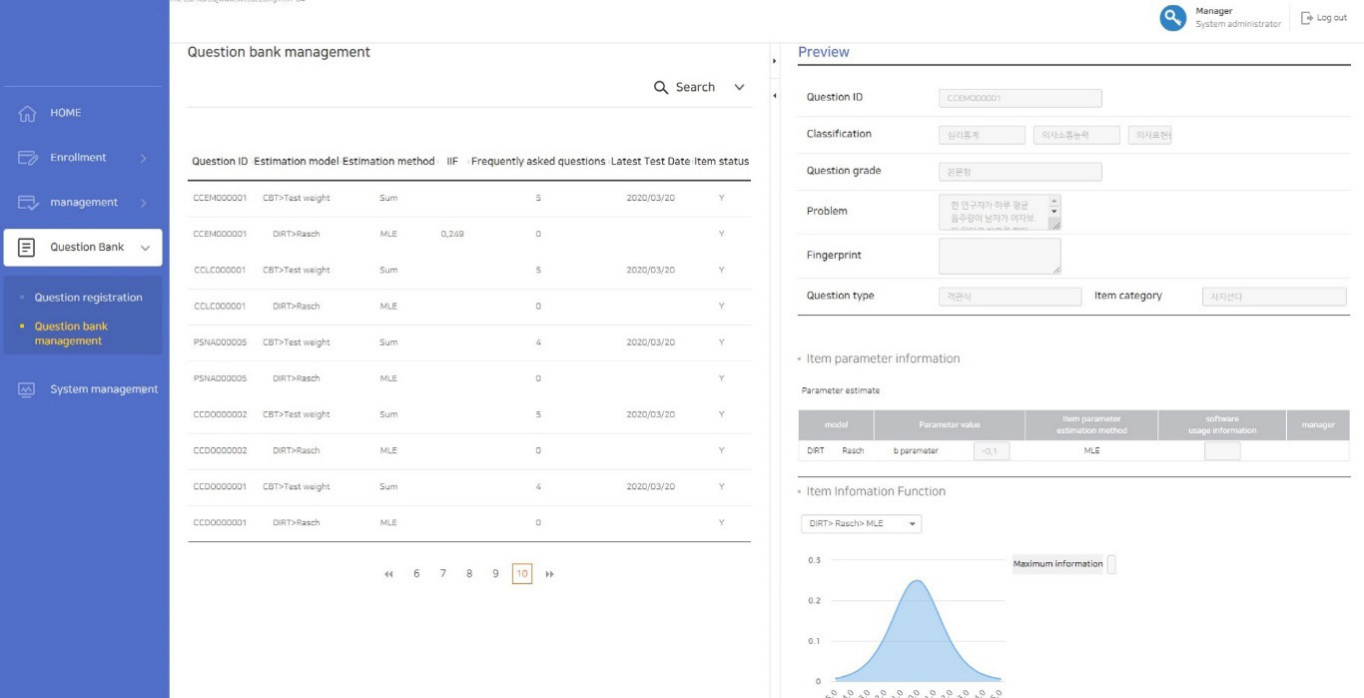
Screenshot of the section for managing items.

In order to establish an item bank, items can be stored on the CAT platform. First of all, basic information about items is added to the item bank such as item category, item type, IRT model (Rasch or 1-, 2-, or 3-parameter logistic models), item parameter estimates, and number of answer options. Multimedia data such as images, audio files, and movies can be uploaded to individual items (Fig. 4). Uploaded items are stored in the item bank, and the item pool should be created to construct an operational test during a specific period. The administrator makes an item pool for each specific test, and cannot select items from the item bank directly to construct a specific test; this prevents errors when the item bank is saved. In addition, the LIVECAT platform provides the item exposure rate, item information, and test information, which are described in the item bank section (Fig. 5).

### Test construction

**Fig. 6.**
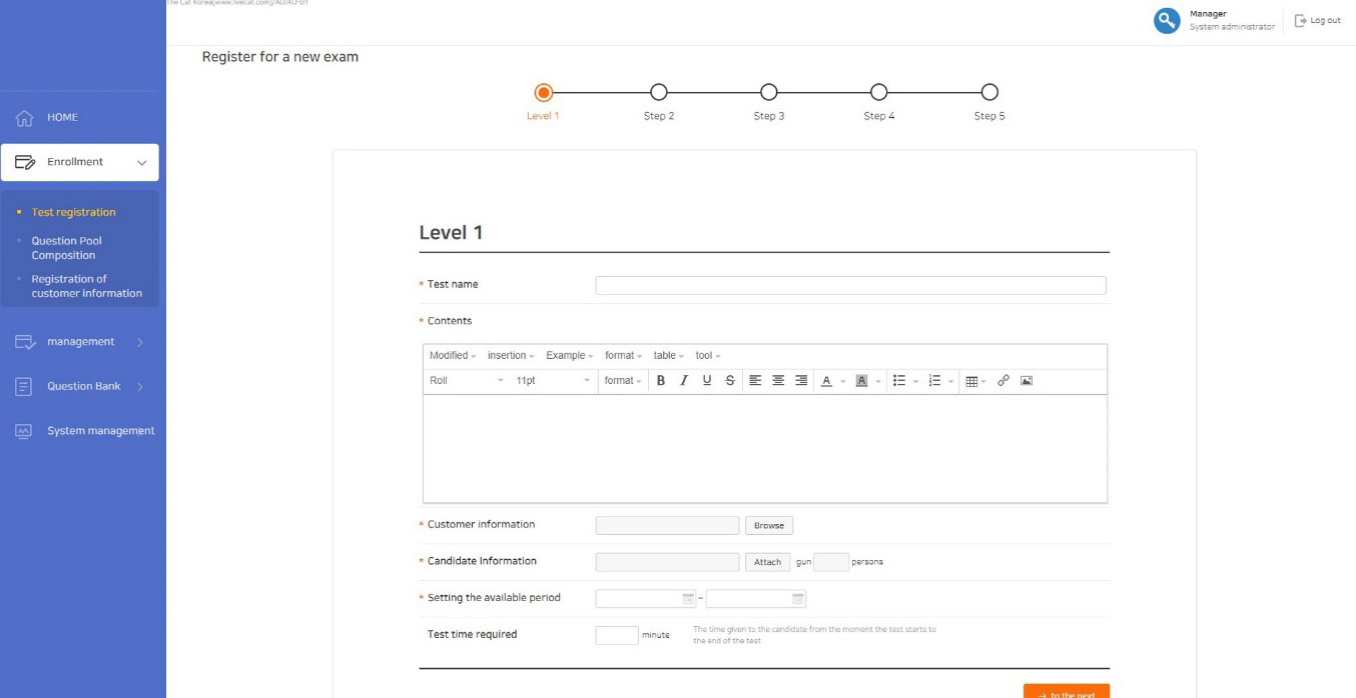
Screenshot of the test information input interface.

In order to construct a test, basic information should be added, such as the title of the test, the start and stop time, the maximum item number, and the minimum item number (Fig. 6). The examinee’s information (i.e., name, ID, and password) is easily uploaded using the spreadsheet form provided by LIVECAT. After inputting basic information for the test, the components of CAT (item selection method, scoring method, termination criterion) must be set. The administrator can choose the components of CAT that he or she wants among the options provided in LIVECAT.

### Components of LIVECAT

LIVECAT provides several options for each component. There are 2 scoring methods in LIVECAT: maximum likelihood estimation (MLE) and expected a posteriori. The MLE method is set as the default.

Of several item selection methods, the LIVECAT platform uses the maximum Fisher information (MFI) method, which selects the next item that has maximum information given the examinee’s ability [3]. The MFI item selection method is applied to every IRT model in LIVECAT.

LIVECAT provides 3 options for the termination criteria of the test (maximum number of items, standard error of estimates, test time period). In addition, the termination criteria can combine 2 or more options. The maximum number of items option causes a test to stop if the number of items administered reaches a prespecified number. The standard error option makes the test stop if the standard error of an examinee’s ability estimate meets a prespecified criterion (e.g., 0.25 or 0.20). The test time period option causes a test to stop if a prespecified test time period is met.

### Test administration and reporting

**Fig. 7.**
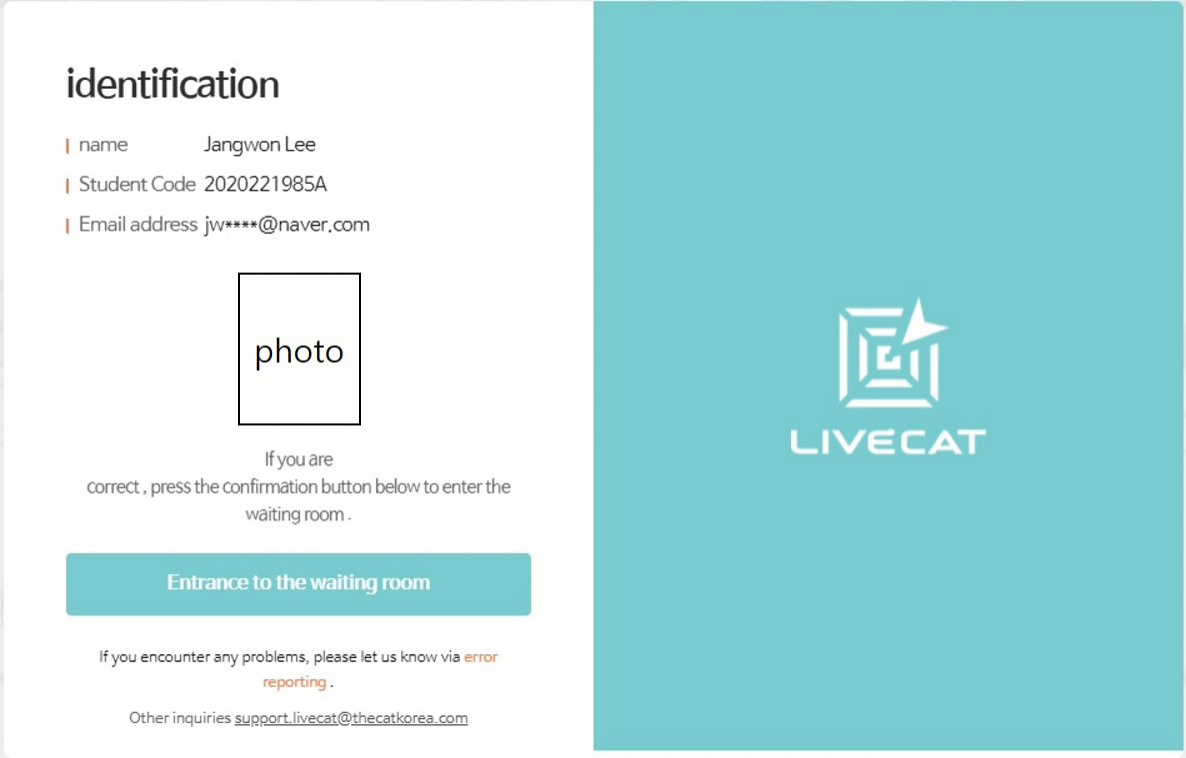
Screenshot of the page containing the examinee’s information.

**Fig. 8.**
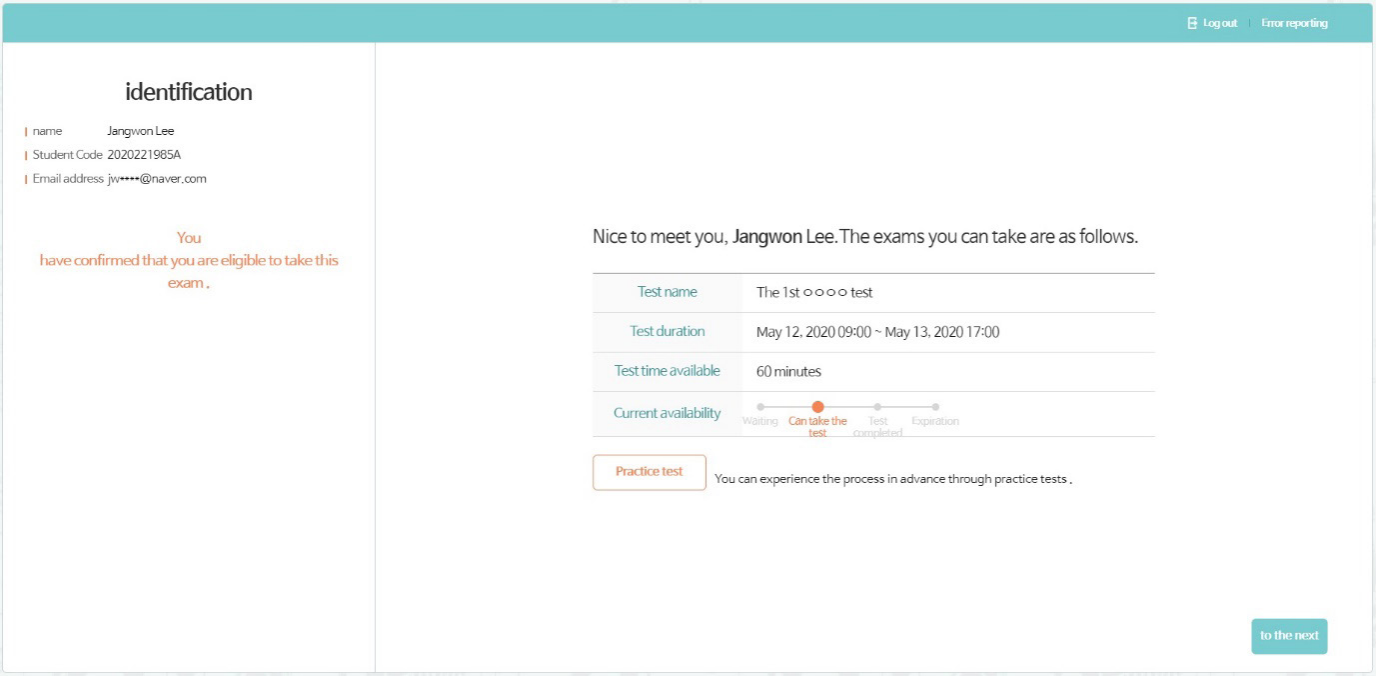
Screenshot of the introduction page for a test.

**Fig. 9.**
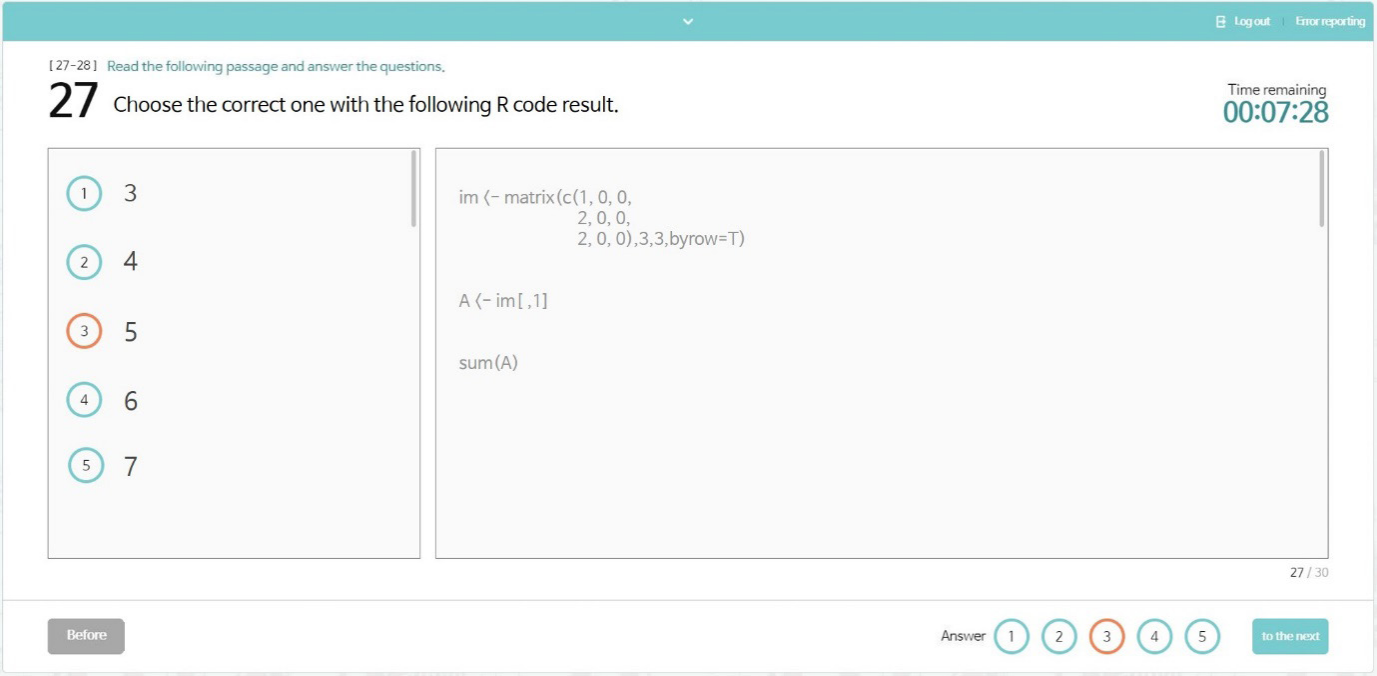
Screenshot of a page that presents an item.

The test administrator can set the test URL in the test construct section. A duplicate URL cannot be used at the same time. Test examinees can take a test by using the test URL. Examinees can take their tests after their ID and password are confirmed (Figs. 7–9). The test can be progressed during the designated time period indicated by the administrator in the test construction section. If an examinee finishes a test, he or she cannot take it again. Since each test is administered through the website, examinees can take tests anywhere without any limitations.

The test results can be reported immediately after a test is completed. LIVECAT provides 3 types of test results: true score, transformed t-score, and pass/fail. Furthermore, each examinee’s responses to each item, the examinee’s ability estimates, and the codes of the items that were presented can be downloaded in spreadsheet form in LIVECAT. Since examinees’ abilities are estimated on a web server, it is unnecessary to score tests by hand.

## Implementation of computerized adaptive testing in the field

The precision and efficiency of LIVECAT have already been demonstrated by implementing a class examination. LIVECAT showed better efficiency than a conventional test. Furthermore, the previous study showed a good correlation between ability estimates obtained from LIVECAT and ability estimates obtained from a conventional test method (r=0.786, P<0.001) [4]. If researchers and test administrators would like to implement the LIVECAT platform in their field, they can obtain a limited version of the LIVECAT platform by completing a request form on the website (http://www.thecatkorea.com/).

### User interface

LIVECAT features an intuitive graphical user interface and provides the above-described test operational functions and several IRT models.

## Conclusion

The LIVECAT platform has several advantages. First, it saves the expenses of preparing and administering tests (e.g., paper costs, printing costs, time). Since LIVECAT is a web-based platform, there is no restriction on place and time. It is also easy to manage item and test information within the LIVECAT platform, which means that a test administrator does not need to be a psychometrician to apply CAT. However, an important consideration when implementing LIVECAT is that the test server should be stable while a test is being taken. In particular, the test server should not be shut down when a high-stakes examination is being administered. An Amazon Web Services (AWS) cloud server can immediately cope with unexpected external problems and keep a test server stable because AWS has infrastructure distributed over the world. The AWS cloud server was adopted to ensure stability for our LIVECAT platform. The recent version of LIVECAT provides only a dichotomous item response model and basic components of CAT. In the near future, LIVECAT will be updated to include advanced functions, such as polytomous item response models, weighted likelihood estimation method, and content balancing method. Once a polytomous item response model is implemented for psychological testing (e.g., personality, attitude), the LIVECAT platform will be used widely for psychological examinations in addition to certification/licensure examinations.

## Supplementary materials

Supplement 1. Audio recording of the abstract.
